# Thermodynamic Assessment of a Solar-Driven Integrated Membrane Reactor for Ethanol Steam Reforming

**DOI:** 10.3390/molecules26226921

**Published:** 2021-11-17

**Authors:** Hongsheng Wang, Bingzheng Wang, Sean-Thomas B. Lundin, Hui Kong, Bosheng Su, Jian Wang

**Affiliations:** 1 MOE Key Laboratory of Hydrodynamic Transients, School of Power and Mechanical Engineering, Wuhan University, Wuhan 430072, China; wanghongsheng@whu.edu.cn; 2 Department of Chemical System Engineering, School of Engineering, The University of Tokyo, 7-3-1 Hongo, Bunkyo-ku, Tokyo 113-8656, Japan; sean@chemsys.t.u-tokyo.ac.jp; 3 Department of Energy Engineering, Zhejiang University, Hangzhou 310027, China; wang_bz@zju.edu.cn; 4 School of Mechanical Engineering, Beijing Institute of Technology University, Beijing 100081, China; 5 Department of Marine Equipment and Mechanical Engineering, Jimei University, Xiamen 361021, China; 6 Fujian Province Key Laboratory of Energy Cleaning Utilization and Development, Xiamen 361021, China; 7 School of Energy and Environment, City University of Hong Kong, Hong Kong, China

**Keywords:** solar thermochemistry, ethanol steam reforming (ESR), mid/low-temperature solar energy, hydrogen permeation membrane (HPM), hydrogen generation, thermodynamic efficiency

## Abstract

To efficiently convert and utilize intermittent solar energy, a novel solar-driven ethanol steam reforming (ESR) system integrated with a membrane reactor is proposed. It has the potential to convert low-grade solar thermal energy into high energy level chemical energy. Driven by chemical potential, hydrogen permeation membranes (HPM) can separate the generated hydrogen and shift the ESR equilibrium forward to increase conversion and thermodynamic efficiency. The thermodynamic and environmental performances are analyzed via numerical simulation under a reaction temperature range of 100–400 °C with permeate pressures of 0.01–0.75 bar. The highest theoretical conversion rate is 98.3% at 100 °C and 0.01 bar, while the highest first-law efficiency, solar-to-fuel efficiency, and exergy efficiency are 82.3%, 45.3%, and 70.4% at 215 °C and 0.20 bar. The standard coal saving rate (SCSR) and carbon dioxide reduction rate (CDRR) are maximums of 101 g·m^−2^·h^−1^ and 247 g·m^−2^·h^−1^ at 200 °C and 0.20 bar with a hydrogen generation rate of 22.4 mol·m^−2^·h^−1^. This study illustrates the feasibility of solar-driven ESR integrated with a membrane reactor and distinguishes a novel approach for distributed hydrogen generation and solar energy utilization and upgradation.

## 1. Introduction

The combustion of fossil fuels worldwide is releasing an enormous quantity of CO_2_ into the atmosphere that is considered largely responsible for environmental problems (e.g., global warming, NO_x_ and SO_x_ pollution). Solar energy is a clean and abundant form of energy with the potential to solve these long-term energy and environmental issues [1]. However, due to the intermittency and low energy density of solar irradiation, solar energy must be converted into stable chemical fuels to create a steady solar energy supply [2]. Hydrogen is considered a green energy carrier, which balances energy density with the capabilities of both long-term storage and ease of conversion to other energy forms [3,4]. Hydrogen can be produced by several solar thermochemical reactions [5,6,7], wherein the fuel reforming process driven by solar energy can generate hydrogen efficiently [8,9].

Among the various fuels, biofuels such as bioethanol are considered to be carbon neutral because the CO_2_ emitted upon combustion was originally absorbed during the growth of the plants [10]. Bioethanol can be produced from secondary biomass, and it has been estimated that more than 442 billion liters of bioethanol can be yielded each year if all the agricultural residues, forestry wastes, and dedicated energy crops are utilized [11,12]. More than 110 billion liters of bioethanol are produced worldwide each year, and the USA and Brazil are the two major producers, producing around 85% of the global bioethanol production [13,14]. Converting bioethanol into hydrogen via solar thermochemical process allows for the simultaneous production of green hydrogen and storage of solar energy. The primary pathway for this is ethanol steam reforming (ESR, shown in Equation (1)) [15]:

(1) $$C_{2}H_{5}\mathrm{OH}(g)+3H_{2}O(g)⇌2{\mathrm{CO}}_{2}(g)+6H_{2}(g),\;\Delta H_{25{\;}^{{}^{\circ}}C}^{⊖}=173.27\;\mathrm{kJ}/\mathrm{mol}$$

One drawback of hydrogen is the complexity and associated cost of transportation over liquid fuels. Thus, on-site hydrogen generation from ESR offers an economic pathway for distributed energy systems, such as refueling stations for hydrogen fuel cell powered vehicles. The H_2_ and CO_2_ products of ESR need to be separated for hydrogen utilization and carbon capture. However, industrial gas separation technologies such as pressure swing adsorption are not sufficiently cost-efficient for small-scale distributed hydrogen refueling stations [16]. Coupling ESR with hydrogen permeation membranes (HPM) in a membrane reactor configuration allows for the selective removal of hydrogen driven by its partial pressure difference between the catalyst bed and the permeate side of the membrane. In addition to single-step ethanol conversion and hydrogen separation, the membrane reactor causes a forward equilibrium shift, which reduces the required reaction temperature and increase the conversion rate. HPMs are made from various materials, including ceramic, polymeric, perovskite, and metallic membranes [17]. Among them, dense metallic membranes offer high selectively and H_2_ permeance. In particular, Pd-Ag membranes are studied because of the ability to inhibit hydrogen embrittlement at lower temperatures [18,19,20]. Pd-Ag membranes are commonly fabricated on porous ceramic supports, which allows them to withstand a high-pressure difference between the two sides of the membrane [21]. Iulianelli et al. had experimentally proved in different research studies that the bioethanol steam reforming in a Pd-based HPM reactor can obtain a higher bioethanol conversion rate than that in a traditional reactor with the catalysts of Co–Pt [22] and Ni/CeO_2_ [23].

The large enthalpy change of ESR and pre-heating of reactants requires a large thermal energy input, which is traditionally provided by fossil fuel combustion leading to additional CO_2_ emissions [19]. With the rapid development of concentrated solar energy (CSE) technologies, solar thermochemical reactions have become an alternative that decreases the consumption of fossil fuels [24,25,26,27]. In most solar thermochemical reactions, solar collectors are used to collect and convert solar energy into thermal energy. Point-focusing solar collectors, such as dish collectors and heliostat field collectors, concentrate sunlight into a point and induce high temperatures in the materials (e.g., 2000 °C) [28]. In contrast, line-focusing parabolic trough solar collectors allow temperatures to be maintained below 500 °C, making them one of the most economical methods of utilizing solar energy [29]. Nevertheless, both types of collectors have attracted increasing attention in recent years. Bai et al. experimentally realized an industrial-scale mid-temperature thermochemical power generation using a structure combining a solar thermal collector and a reactor for methanol synthesis [30]. Wang et al. [31] modeled a solar dish collector integrated with HPM for non-oxidative methane dehydroaromatization in the temperature range of 600 °C to 800 °C and obtained a theoretical energy efficiency of 85.9%. Tou et al. [32] experimentally demonstrated the single-step continuous splitting of CO_2_ into separate streams of CO and O_2_ under steady-state isothermal/isobaric conditions using a solar-driven ceria membrane reactor. Operation at 1600 °C, 3 × 10^−6^ bar *P*_O2_, and 3500 sun radiation resulted in an O_2_ separation rate of 0.024 μmol·s^−1^·cm^−2^. Giaconia [33] studied methane steam reforming using a membrane reactor driven by concentrated solar power and achieved a conversion rate twice that of a conventional reformer operating at thermodynamic equilibrium. He et al. [5] modeled propane dehydrogenation using a trough solar collector combined with a membrane reactor and observed a maximum propane conversion rate and propylene yield of 99.2% and 98.3% at a reaction temperature of 400 °C and permeate pressure of 10^−5^ bar. In each study, the presence of the membrane reactor enhances efficiencies as compared with the use of traditional reactors without membrane.

Although the kinetics and catalytic processes of ESR have been widely studied [34], the thermodynamic and environmental performance of a solar-driven ESR membrane reactor have not been researched. Thus, a novel solar-driven ESR system combined with an HPM reactor is proposed and analyzed. This system allows for the conversion of intermittent low-grade solar thermal energy into high-grade chemical energy. The thermodynamic efficiencies and conversion rates are optimized and compared with those using a traditional reactor. The carbon dioxide emissions reduction rate (CDRR) and standard coal savings rate (SCSR) were also calculated to gauge the environmental performance of this system.

## 2. System Description

**Figure 1:**
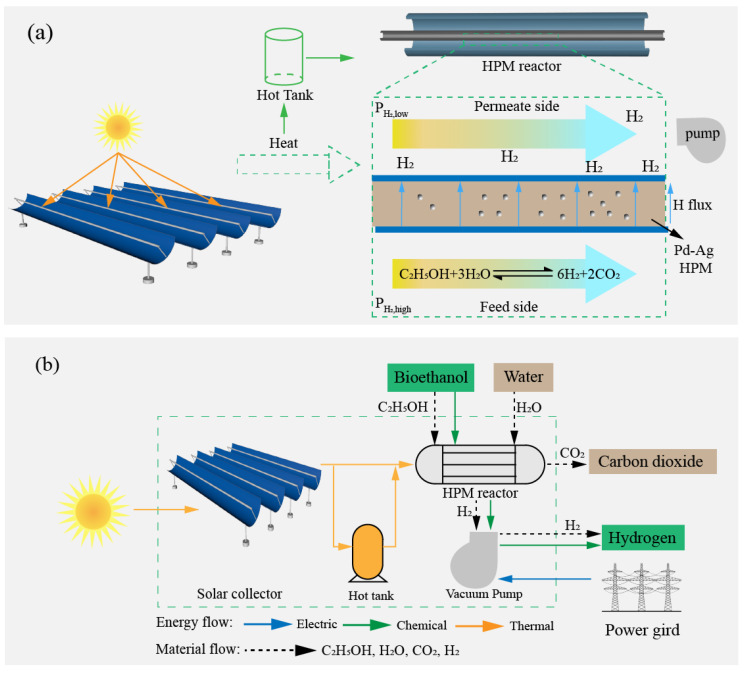
Conceptual schematic (**a**) and system boundary containing input/output flows (**b**) of a solar-driven ESR membrane reactor.

A conceptual schematic of a solar-driven ESR membrane reactor is shown in Figure 1a. Heat energy is collected from solar irradiation by a trough solar collector, which subsequently is either used directly to drive the ESR membrane reactor or stored for later use in a hot tank. The ESR membrane reactor consists of a packed-bed tube-in-shell configuration. The exterior shell is impermeable, while the interior tube is an HPM consisting of a supported Pd-Ag composite membrane. The packed-bed (feed side) consists of a commercial nickel-based catalyst [35] to catalyze the ESR reaction, while the permeate side is connected to a vacuum pump that maintains a pressure conducive for hydrogen separation.

Figure 1b shows the main input/output flows of the system. The input water and ethanol are preheated to the reaction temperature by solar thermal energy before being fed at the stoichiometric molar ratio of H_2_O:C_2_H_5_OH = 3:1, and the high purity hydrogen and carbon dioxide are the system material output. Solar energy, chemical energy of ethanol, and electric energy are the input energy of the system, and chemical energy of hydrogen is the system output energy. In the reaction process, generated hydrogen permeates through the HPM driven by the pressure difference between the feed and permeate sides, which increases the conversion rate of ESR and purifies the hydrogen. The main simulation work is completed by Python 3.6.6, and the required thermodynamic parameters are provided by HSC software. The thermodynamic results are obtained based on the following assumptions:

(1) Steady-state operation of the ESR membrane reactor is assumed [36];
(2) Pressure-drop across the catalyst bed is negligible [37];
(3) Ideal plug-flow operation is assumed (i.e., gas diffusion is negligible) [38].

## 3. Theoretical Formulations

Thermodynamic efficiencies offer insight into the capability of a system to convert and utilize solar energy and are thus systematically analyzed. Herein, the first-law thermodynamic efficiency, solar-to-fuel efficiency, and exergy efficiency are chosen as three key indicators for gauging the thermodynamic performance of the system. The first-law thermodynamic efficiency (*η*_HHV_, Equation (2)) is the ratio of system energy output to energy input, and is expressed as [39]

(2) $$\eta_{\mathrm{HHV}}=\frac{n_{H_{2}}\cdot HHV_{H_{2}}}{\eta_{\mathrm{opt}}^{-1}\eta_{\mathrm{abs}}^{-1}\cdot (Q_{\mathrm{preheat}}+Q_{\mathrm{enthalpy}})+\eta_{s\to e}^{-1}\cdot W_{p}+n_{E}\cdot HHV_{E}}$$

where *n*_H_2__ and *n*_E_ are the molar amount of generated hydrogen and consumed ethanol; *HHV*_H_2__ and *HHV*_E_ are the molar higher heating value of hydrogen and ethanol, taken as 286 kJ·mol^−1^ and 1410 kJ·mol^−1^ [40]; *η*_opt_ is the optical efficiency of the trough solar collector, taken as 73% [41]; *η*_abs_ exhibited by Equation (3) is the absorption efficiency of the collector, expressed as [42]

(3) $$\eta_{\mathrm{abs}}=\alpha -\frac{\varepsilon \cdot \sigma \cdot {\left(T_{H}+273.15\right)}^{4}}{DNI\cdot C_{\mathrm{collector}}}$$

where *α* and *ε* are the absorptivity and emissivity of the collector, taken as 0.9 and 0.1 [42]; *σ* is Stefan–Boltzmann’s constant; *T*_H_ is the reaction temperature; *DNI* is the direct normal irradiation; *C*_collector_ is the concentration ratio of the collector; *η*_s→e_ is the solar-to-electric efficiency, taken as 15% (commercial photovoltaic (PV) cell efficiency) and 40% (multiple-junction GaAs PV efficiency in laboratory). *Q*_preheat_ is the heat consumed to raise the temperatures of ethanol and water from room temperature to reaction temperature and is calculated as Equation (4) [31,40]:

(4) $$\begin{array}{l} Q_{\mathrm{preheat}}=n_{E,\mathrm{init}}\cdot (\int_{T_{L}}^{78{\;}^{{}^{\circ}}C}C_{p,C_{2}H_{5}\mathrm{OH}(l)}dT+39.234+n_{E,\mathrm{init}}\cdot \int_{78{\;}^{{}^{\circ}}C}^{T_{H}}C_{p,C_{2}H_{5}\mathrm{OH}(g)}dT)\\ +n_{W,\mathrm{init}}\cdot (\int_{T_{L}}^{100{\;}^{{}^{\circ}}C}C_{p,H_{2}O(l)}dT+40.873+\int_{100{\;}^{{}^{\circ}}C}^{T_{H}}C_{p,H_{2}O(g)}dT) \end{array}$$

where *n*_E,init_ and *n*_H_2_O,init_ are the initial molar amounts of ethanol and water; *C*_p,E_ and *C*_p,H_2_O_ are the specific heat capacities of ethanol and water; 39.234 and 40.873 (kJ·mol^−1^) are the molar vaporization latent heat of ethanol and water [40]. *Q*_enthalpy_ is the heat consumed by the enthalpy change of ESR reaction; *W*_P_ is the exergy consumed by the vacuum pump to maintain a low pressure for hydrogen separation, and expressed as Equation (5):

(5) $$W_{P}=n_{H_{2},\mathrm{out}}\cdot RT_{0}\ln\left(P_{0}/P_{H_{2},\mathrm{out}}\right)$$

where *n*_H_2_,out_ is the molar amount of separated hydrogen; *R* is universal gas constant, taken as 8.314 J·mol^−1^·K^−1^; *T*_0_ is the room temperature, given as 25 °C; *P*_0_ is the reaction pressure, fixed at 1 bar in this research; *P*_H_2_,out_ is the permeate pressure. In thermodynamic equilibrium state, the hydrogen partial pressure should be equal on two sides of the HPM, and thus the *n*_H_2_,out_ can be derived as Equation (6):

(6) $$n_{H_{2},\mathrm{out}}=\frac{2\cdot n_{E,\mathrm{init}}\cdot \left(3\cdot \alpha_{0}\cdot P_{0}-2\cdot \alpha_{0}\cdot P_{H_{2},\mathrm{out}}-2\cdot P_{H_{2},\mathrm{out}}\right)}{P_{0}-P_{H_{2},\mathrm{out}}}$$

where *α*_0_ is the conversion rate of ESR.

The *η*_HHV_ can exhibit the total energy conversion and utilization capability of this system. While the energy input in *η*_HHV_ calculation contains the chemical energy of ethanol, to eliminate the influence of chemical energy in reactants and evaluate the conversion from solar energy to chemical energy in products, the solar-to-fuel efficiency (*η*_s→f_), which is defined as the ratio of chemical energy increment to solar energy input, is expressed as Equation (7):

(7) $$\eta_{s\to f}=\frac{n_{H_{2}}\cdot HHV_{H_{2}}-n_{E}\cdot HHV_{E}}{\eta_{\mathrm{opt}}^{-1}\eta_{\mathrm{abs}}^{-1}\cdot (Q_{\mathrm{preheat}}+Q_{\mathrm{enthalpy}})+\eta_{s\to e}^{-1}\cdot W_{p}}$$

In Equations (2) and (7), *W*_P_, the consumed vacuum pump exergy (theoretical minimum energy), is used to calculate the upper bound of thermodynamic efficiencies and exhibits the potential of this system in further application. The vacuum pump efficiency is the ratio of exergy output to electricity input of the vacuum pump, calculated as Equation (8) [43,44]:

(8) $$\eta_{p}={\left(\frac{P_{H_{2},\mathrm{out}}}{P^{⊖}}\right)}^{0.544}$$

where *P*^⊖^ is the standard pressure. After taking the vacuum pump efficiency into consideration, the first-law efficiency and solar-to-fuel efficiency with real separation energy (*η*_HHV,real_ and *η*_s→f,real_) can be expressed as Equations (9) and (10):

(9) $$\eta_{\mathrm{HHV},\mathrm{real}}=\frac{n_{H_{2}}\cdot HHV_{H_{2}}}{\eta_{\mathrm{opt}}^{-1}\eta_{\mathrm{abs}}^{-1}\cdot (Q_{\mathrm{preheat}}+Q_{\mathrm{enthalpy}})+\eta_{s\to e}^{-1}\eta_{p}^{-1}\cdot W_{p}+n_{E}\cdot HHV_{E}}$$

(10) $$\eta_{s\to f,\mathrm{real}}=\frac{n_{H_{2}}\cdot HHV_{H_{2}}-n_{E}\cdot HHV_{E}}{\eta_{\mathrm{opt}}^{-1}\eta_{\mathrm{abs}}^{-1}\cdot (Q_{\mathrm{preheat}}+Q_{\mathrm{enthalpy}})+\eta_{s\to e}^{-1}\eta_{p}^{-1}\cdot W_{p}}$$

Equations (2), (7), (9) and (10) mainly focus on the energy conversion amount, while the conversion efficiency of energy quality is also significant. The exergy efficiency (*η*_ex_), which is the ratio of exergy output to exergy input, is defined as Equation (11) [39,45]:

(11) $$\eta_{\mathrm{ex}}=\frac{n_{H_{2}}\cdot Ex_{H_{2}}}{Ex_{\mathrm{solar}}+n_{E}\cdot Ex_{E}}$$

(12) $$Ex_{\mathrm{solar}}=\left(1-\frac{4T_{0}}{3T_{\mathrm{sun}}}+\frac{1}{3}\cdot {\left(\frac{T_{0}}{T_{\mathrm{sun}}}\right)}^{4}\right)\cdot \left(\left(Q_{\mathrm{preheat}}+Q_{\mathrm{enthalpy}}\right)\cdot \eta_{\mathrm{opt}}^{-1}\eta_{\mathrm{abs}}^{-1}+\eta_{s\to e}^{-1}\eta_{p}^{-1}\cdot W_{p}\right)$$

where *Ex*_H_2__ and *Ex*_E_ are the chemical exergies of hydrogen and ethanol, taken as 235 kJ·mol^−1^ and 1308 kJ·mol^−1^; *Ex*_solar_ calculated by Equation (12) is the input exergy of solar energy.

In addition to the thermodynamic performance, the environmental performance is also considered in terms of the fossil fuel saved (SCSR, Equation (13)) and CO_2_ emissions reduced (CDRR, Equation (14)). Assuming the absorbed solar energy is provided by standard coal, the SCSR and CDRR can be defined as

(13) $$\mathrm{SCSR}=\frac{\eta_{c\to h}^{-1}({\dot{Q}}_{\mathrm{preheat}}+{\dot{Q}}_{\mathrm{enthalpy}})+\eta_{c\to e}^{-1}\cdot \eta_{p}^{-1}\cdot {\dot{W}}_{p}}{q_{\mathrm{coal}}}$$

(14) CDRR=μ·SCSR

where *η*_c→h_ and *η*_c→e_ are conversion efficiencies from standard coal to heat and electricity, which are taken as 80% and 40% [46,47]; *q*_coal_ is the heating value of standard coal, taken as 2.931 × 10^4^ kJ·kg^−1^; and *μ* is the mass ratio of carbon dioxide emissions to standard coal combustion, taken as 2.45 [48].

## 4. Results and Discussion

**Figure 2:**
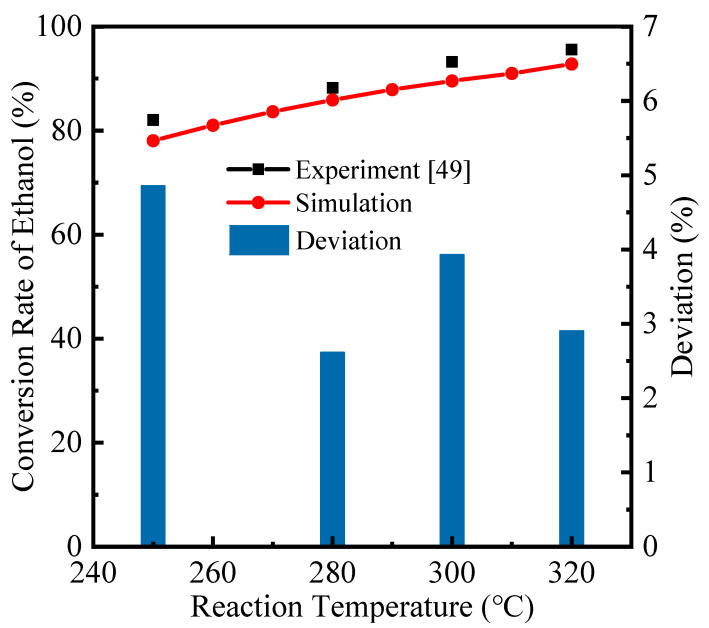
Comparison between theoretical and experimental ethanol conversion rate of ESR for model validation.

The proposed system was investigated and analyzed by numerical simulation and calculation. With a feed pressure of 1 bar, the thermodynamic and environmental performances were studied for temperatures of 100 °C to 400 °C and permeate pressures of 0.01 bar to 0.75 bar. To verify the simulated reaction results, the conversion rate of ethanol in this research is compared with the experimental results [49], which are under the temperature range of 250–320 °C and reaction pressure of 1 bar without hydrogen separation, shown in Figure 2. The experimental conversions are slightly higher than the thermodynamic results in this simulation due to the potential side reactions (e.g., ethanol splitting), while the deviation is smaller than 5%, which denotes that the simulation results fit well with those from the experiment.

**Figure 3:**
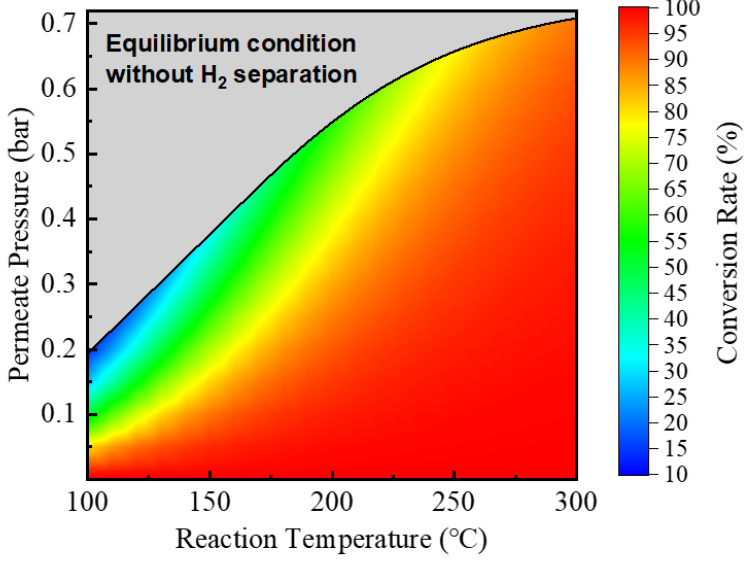
ESR conversion rate at different reaction temperatures and permeate pressures.

Figure 3 exhibits the conversion rate of ESR under different reaction temperatures and permeate pressures. The endothermicity of ESR means that the thermodynamic equilibrium conversion will increase with increasing temperature. Decreasing permeate pressure increases the removal of the generated hydrogen by the HPM reactor, which then increases the conversion rate of ESR due to Le Chatelier’s principle. However, the grey zone in Figure 3 represents the region wherein the permeate pressure is higher than the hydrogen partial pressure in equilibrium, meaning that the HPM has no driving force and cannot remove the generated hydrogen. The upper bound of the colored area denotes the equilibrium-limited conversion rate without hydrogen separation. At 100 °C, the equilibrium conversion rate of a traditional reactor is 14.8%, and the corresponding hydrogen partial pressure is 0.19 bar. By lowering the permeate pressure to 0.01 bar, the generated hydrogen can be removed, causing the conversion rate to increase to 98.3%. At 300 °C, however, the traditional reactor already results in an equilibrium conversion of 89.6%, so the presence of the HPM and low permeate pressure has a lower effect. Operating at higher temperatures consumes more thermal energy and separation work with less increase in conversion, which negatively influences the thermodynamic efficiencies of the system.

**Figure 4:**
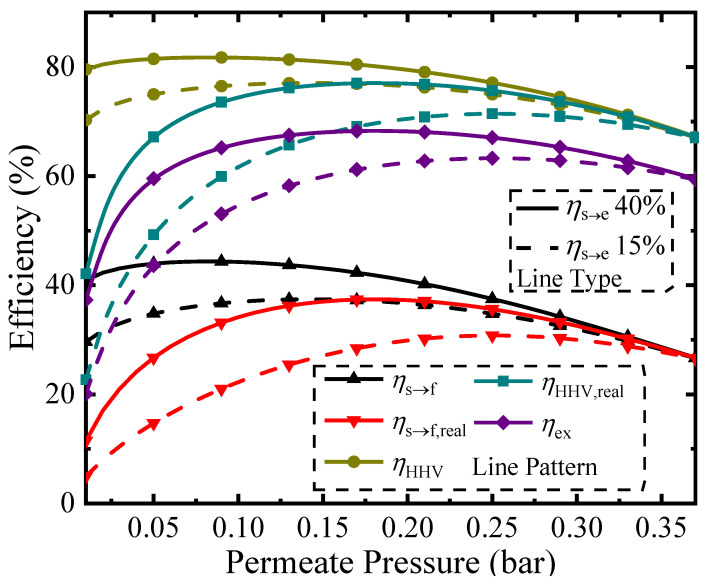
Thermodynamic efficiency versus permeate pressure at a reaction temperature of 150 °C.

Figure 4 shows various thermodynamic efficiencies (Equations (2), (7), (9), (10) and (11)) under different permeate pressures at a temperature of 150 °C. The line type distinguishes different solar-to-electric efficiencies (*η*_s→e_ of 15% or 40%). The upper bound efficiencies are given when separation exergy is used (*η*_HHV_ and *η*_s→f_), whereas the best efficiencies using common separation methods such as vacuum pumping are calculated using real separation energy (*η*_HHV,real_ and *η*_s→f,real_). The difference between these values is the potential for improvement in efficiency if the separation method is improved.

Permeate pressure affects the pump efficiency (*η*_p_), hydrogen separation exergy (*W*_P_), and conversion rate, which leads to an optimum efficiency at some specific permeate pressure for each calculation. This is the result of two different competing influences. As shown in Figure 3, increasing the permeate pressure decreases the conversion rate, which results in lower solar energy conversion into chemical energy and a corresponding negative influence on efficiencies. Simultaneously, the pump efficiency is increased at higher permeate pressures, which reduces the required separation exergy and corresponding separation energy of the vacuum pump. Optimization of efficiencies using separation exergy and *η*_s→e_ = 40% results in maximums for *η*_HHV_, *η*_s→f_, and *η*_ex_ of 81.8%, 44.4%, and 64.2% at a permeate pressure of 0.08 bar; optimization of efficiencies using real separation energy results in maximums for *η*_HHV,real_ and *η*_s→f,real_ of 77.1% and 37.4% at a permeate pressure of 0.18 bar.

**Figure 5:**
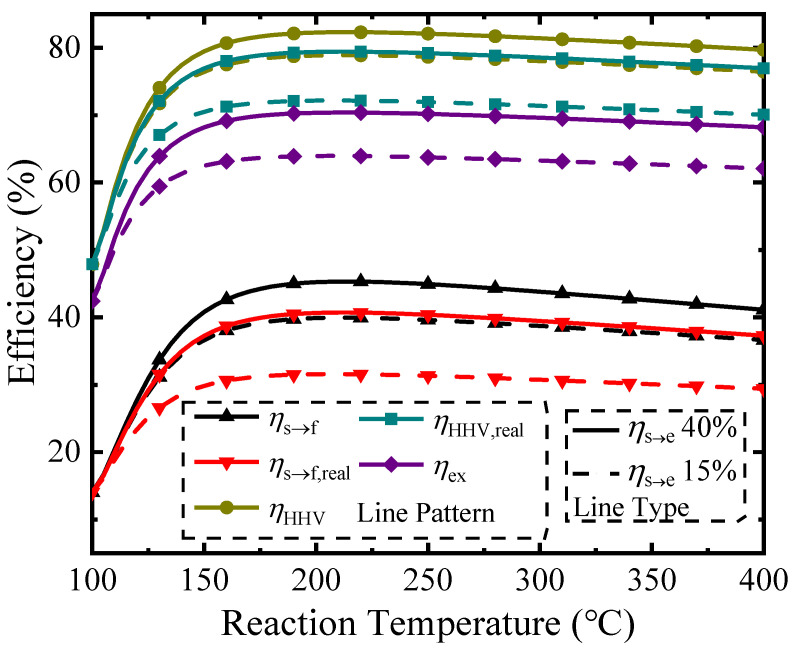
Thermodynamic efficiency versus temperature for a permeate pressure of 0.2 bar.

In addition to the permeate pressure, reaction temperature has a significant effect on performance. Because Figure 3 shows that a permeate pressure of 0.2 bar can separate hydrogen at nearly all temperatures, the permeate pressure was set to 0.2 bar, and the thermodynamic efficiencies were calculated for temperatures of 100 °C to 400 °C (Figure 5). The reaction temperature affects the preheating energy, enthalpy change, and conversion rate. As the temperature increases, all efficiencies initially increase and then decrease. This efficiency maximum occurs because the increase in conversion with increasing temperature is balanced by an increase in energy consumption to preheat the reactants. With the *η*_s→e_ of 40%, these effects balance at a temperature of 215 °C using separation exergy, resulting in maximum thermodynamic efficiencies for *η*_HHV_, *η*_s→f_, and *η*_ex_ with separation exergy of 82.3%, 45.3%, and 70.4%. The corresponding maximum using separation energy occurs at 210 °C, resulting in *η*_HHV,real_ and *η*_s→f,real_ of 79.4% and 40.7%.

**Figure 6:**
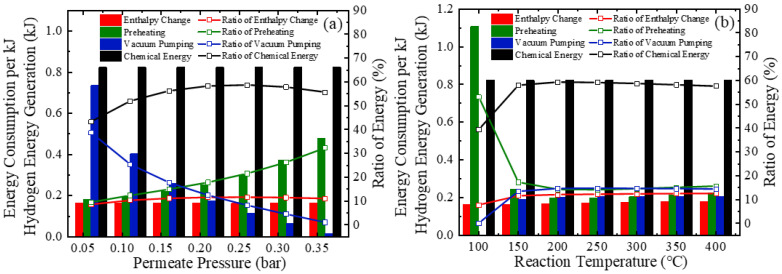
Energy consumption per kJ hydrogen energy generated (*η*_s→e_ = 15%) under (**a**) different permeate pressures (reaction temperature of 150 °C) and (**b**) different reaction temperatures (permeate pressure of 0.2 bar).

While the analyses of Figure 3 and Figure 4 are useful for a qualitative determination of system efficiencies, an analysis concerning the change in efficiency versus kJ of hydrogen energy formed is necessary for a quantitative assessment of the proposed system. Figure 6 exhibits the energy consumption variation per kJ of hydrogen energy generated using real separation energy and *η*_s→e_ of 15%. The energy consumption consists of the enthalpy change, preheating, vacuum pump, and chemical energy of reactants. The chemical energy consumption is assumed to be constant due to the fixed stoichiometric ratio in this research.

In Figure 6a the preheating energy increases with increasing permeate pressure because increasing permeate pressure reduces conversion and results in a larger feed rate to be preheated per kJ hydrogen energy. Simultaneously, the energy consumed by the vacuum pump decreases due to a decrease in the separation exergy (*W*_P_) and increase in pump efficiency (*η*_p_). This results in an optimal permeate pressure of c.a. 0.25 bar.

The energy consumption dependence with reaction temperature is shown in Figure 6b. The enthalpy change of Equation (1) has a slight increase from 100 °C to 400 °C (179 kJ·mol^−1^ to 197 kJ·mol^−1^), and thus the energy consumed of enthalpy change goes up. At any permeate pressure, the pump efficiency is constant and the quantity of hydrogen separated per kJ hydrogen energy generated can be expressed as Equation (6) divided by the amount of hydrogen generated, shown in Equation (15) as follow:

(15) $$n_{H_{2},\mathrm{out},\mathrm{per}}=\frac{\left(3\cdot P_{0}-2\cdot P_{H_{2},\mathrm{out}}-\frac{2\cdot P_{H_{2},\mathrm{out}}}{\alpha_{0}}\right)}{3\cdot (P_{0}-P_{H_{2},\mathrm{out}})}$$

This indicates that the conversion rate (*α*_0_) increases along with rising temperature, meaning the pump work increase slightly due to the increase in separated hydrogen, as shown in Figure 6b. Note that at 100 °C the hydrogen partial pressure in equilibrium is slightly less than 0.2 bar (Figure 3), so the consumed vacuum pump energy is zero. The energy required for preheating shows a maximum at c.a. 200 °C because of opposing influences arising from conversion rate and reaction temperature.

**Figure 7:**
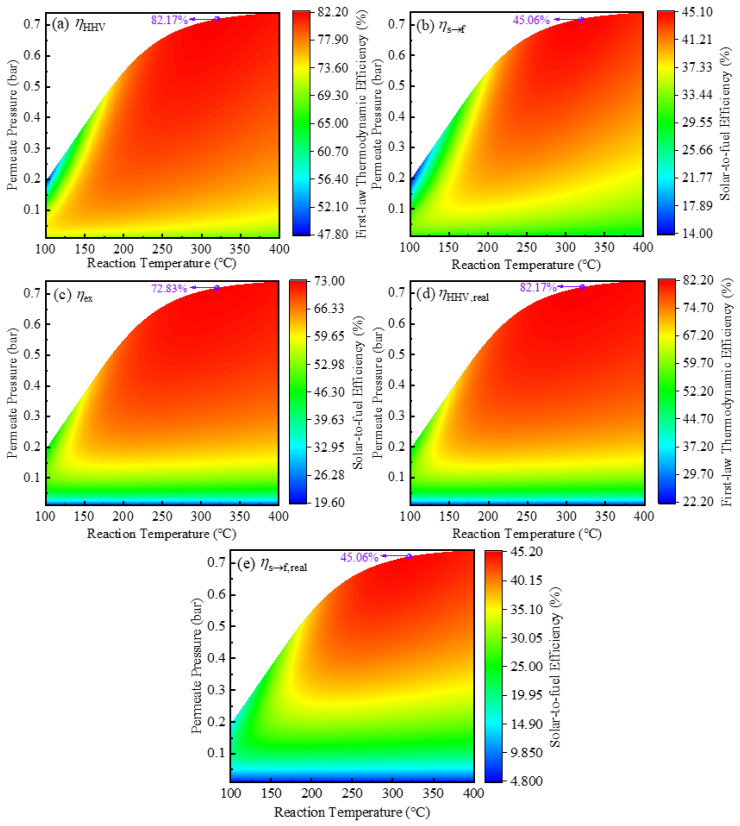
Thermodynamic efficiencies containing (**a**) *η*_en_, (**b**) *η*_s→f_, (**c**) *η*_ex_, (**d**) *η*_en,real_, and (**e**) *η*_s→f,real_ versus temperature and permeate pressure. The maximum efficiency has been marked in the subfigures as purple points.

The thermodynamic efficiency under different temperatures and permeate pressures is shown in Figure 7. In the ESR membrane reactor, the hydrogen is generated and purified in a single step, which increases conversion and eliminates the requirement for a separate separation process downstream. At higher temperatures (e.g., greater than 280 °C in Figure 7a–e), the optimal efficiencies are obtained in the absence of hydrogen separation. This is because the use of a vacuum pump leads to additional energy consumption, while the conversion rate cannot be significantly improved due to the already high thermodynamic limitation of ESR at high temperatures. The highest *η*_HHV_, *η*_s→f_, and *η*_ex_ can be 82.17%, 45.06%, and 72.83% at 320 °C without hydrogen separation. Instead, the solar-driven ESR membrane reactor is beneficial at lower temperatures due to increased efficiencies, which results in lower requirements for irradiation intensity and longer working hours. In the case of higher irradiation intensity, the reactant flow rate can be increased to obtain a higher yield.

**Figure 8:**
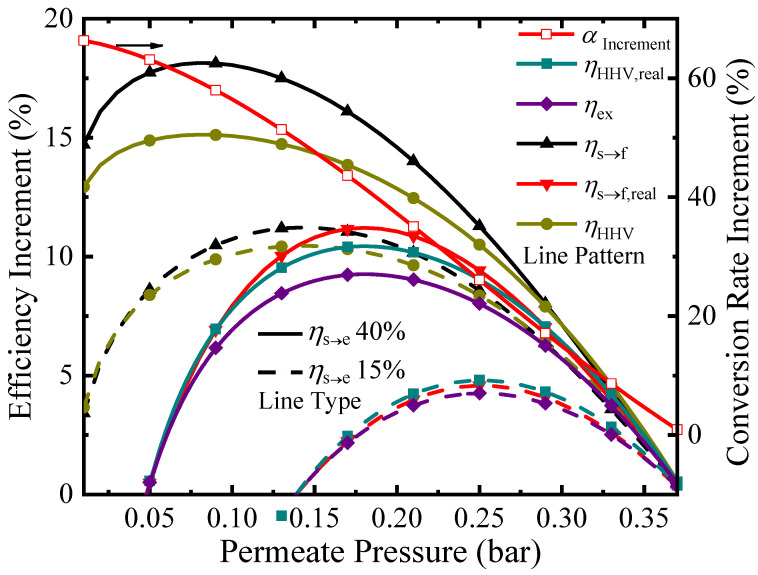
Increment of thermodynamic efficiency and conversion rate compared with those in a traditional reactor versus permeate pressure at a reaction temperature of 150 °C.

To clearly illustrate the benefits of the proposed ESR membrane reactor over a traditional reactor without hydrogen separation, a plot of the efficiency improvement increments versus permeate pressure at 150 °C is shown in Figure 8. At 150 °C, the equilibrium pressure of hydrogen is 0.37 bar, so the membrane reactor only improves the system for permeate pressures below this level. The conversion increases with decreasing permeate pressure due to the increasing driving force for hydrogen separation, with a maximum increase of 66.3% achieved at a permeate pressure of 0.01 bar. However, according to Equations (5) and (8), the increase in exergy and decrease in pump efficiency causes a maximum in the thermodynamic efficiencies to appear. Regarding the separation exergy, the improvement over a traditional reactor for *η*_HHV_, *η*_s→f_, and *η*_ex_ can achieve 15.1%, 18.1%, and 5.21% at 0.08 bar with *η*_s→e_= 40%, respectively. As for the efficiencies regarding real separation energy, the *η*_HHV,real_ and *η*_s→f,real_ can achieve maximum increases of 10.4% and 11.2% at 0.18 bar and *η*_s→e_ of 40%.

**Figure 9:**
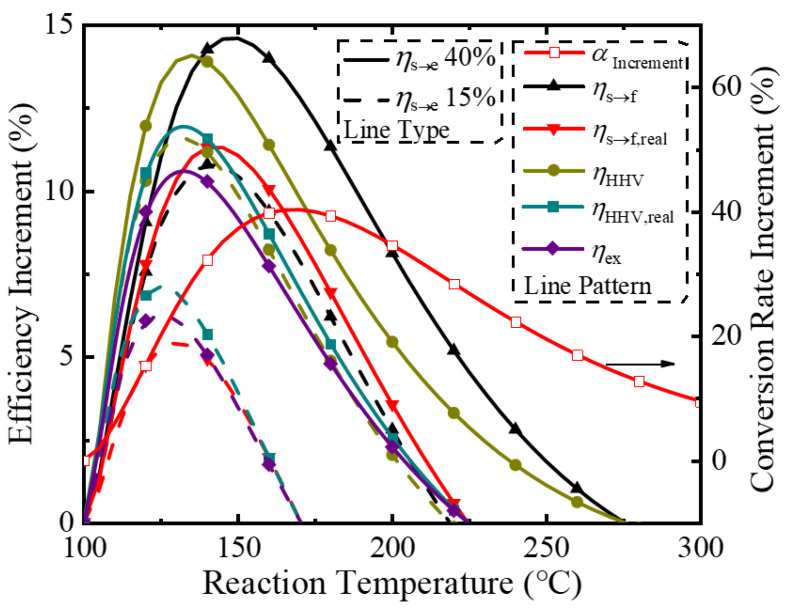
Increment of thermodynamic efficiency and conversion rate compared with those in a traditional reactor versus reaction temperature for a permeate pressure of 0.2 bar.

Figure 9 exhibits the increment of thermodynamic efficiency and conversion rate compared with those in a traditional reactor under different reaction temperatures at a permeate pressure of 0.2 bar. At 100 °C, the hydrogen partial pressure at equilibrium condition in a traditional reactor is 0.19 bar, so the membrane reactor with a permeate pressure of 0.2 bar cannot improve the operation. However, as the temperature increases, the hydrogen partial pressure increases. An increased hydrogen partial pressure allows for a faster removal of hydrogen using a membrane, which causes an increase in the conversion. The largest conversion increase is 40.3% at 170 °C, whereupon further temperature increases observe a decrease in conversion improvement due to the limited improvement potential from an already high thermodynamic limitation of ESR, coupled with an increase in energy consumption of preheating and enthalpy change. With *η*_s→e_ = 40%, the *η*_s→f,real_ has a maximum increment of 11.3% at 140 °C, and the *η*_HHV,real_ has a maximum increment of 12.0% at 130 °C.

**Figure 10:**
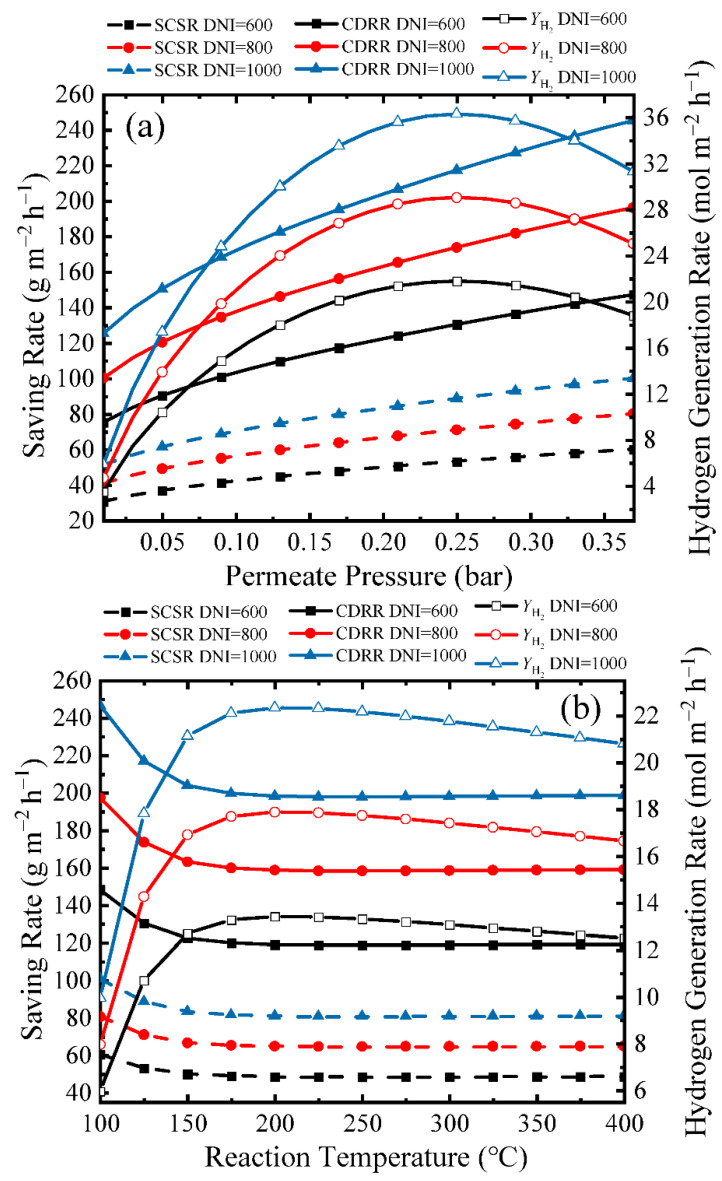
Variation of SCSR, CDRR, and *Y*_H_2__ with real separation energy and *η*_s→e_ of 15% versus (**a**) permeate pressure at a reaction temperature of 150 °C, (**b**) reaction temperature at a permeate pressure of 0.2 bar.

In addition to the thermodynamic performance, environmental performance is also vital for the evaluation of this system. Variation of SCSR, CDRR, and hydrogen generation rate (*Y*_H_2__) with real separation energy and *η*_s→e_ of 15% under different working conditions are shown in Figure 10. Solar energy is collected and stored in a hot tank to supply heat to the ESR membrane reactor. The *DNI* determines the amount of available energy. Figure 10a exhibits the SCSR, CDRR, and *Y*_H_2__ with real separation energy under different permeate pressures at 150 °C. The amount of input energy at a certain *DNI* is constant, and thus a higher *η*_s→f,real_ shown in Figure 4 can reach a higher *Y*_H_2__. The highest *Y*_H_2__ is 36.3 mol·m^−2^·h^−1^ at 0.25 bar, *DNI* of 1000 W·m^−2^. In the calculation of SCSR, the *η*_c→e_ and *η*_s→e_ are taken as 40% and 15%, and thus when energy is used to generate electricity, 1 kJ solar energy can be considered as equal to 0.375 kJ (15%/40%) chemical energy of standard coal. The *η*_c→h_ is 80%, and the conversion efficiency of solar to heat can be considered as the product of optical efficiency and absorption efficiency, which is about 65.7% in this research, and thus 1 kJ solar energy is equivalent to 0.821 kJ (65.7%/80%) chemical energy of standard coal when the energy is used to generate heat. Thus, increasing the proportion of solar energy consumed in the form of thermal energy results in increases in SCSR and CDRR based on Equation (13). The highest *Y*_H_2__ at *DNI* of 1000 W·m^−2^ results in an SCSR and CDRR of 88.7 g·m^−2^·h^−1^ and 217 g·m^−2^·h^−1^. In Figure 10b, the trends of SCSR, CDRR, and *Y*_H_2__ are the same as those in Figure 10a. The highest *Y*_H_2__ at a *DNI* of 1000 W·m^−2^ (22.4 mol·m^−2^·h^−1^) is obtained at 200 °C, with a corresponding SCSR and CDRR of 101 g·m^−2^·h^−1^ and 247 g·m^−2^·h^−1^.

**Figure 11:**
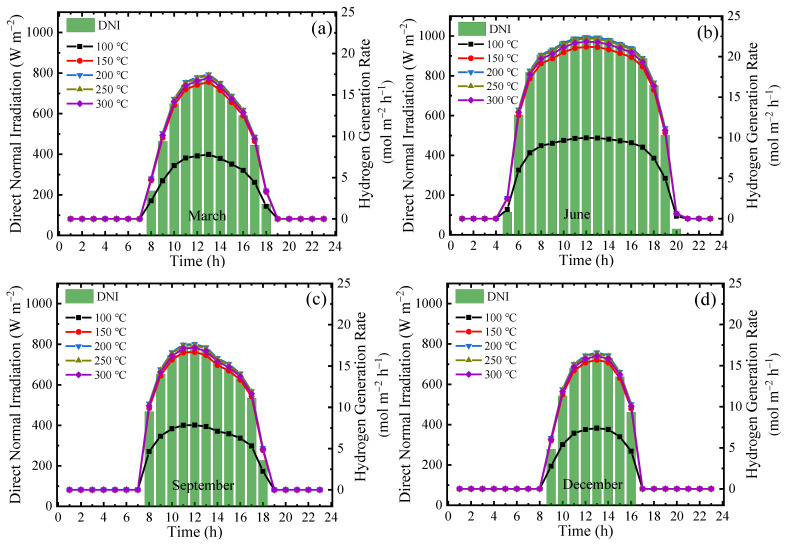
Variation in *DNI* and *Y*_H_2__ on a typical sunny day of different months for different reaction temperatures at a permeate pressure of 0.2 bar with real separation energy (*η*_s→e_ = 15%): (**a**) March; (**b**) June; (**c**) September; and (**d**) December.

To evaluate the system performance at a practical scene, the hourly *DNI* and corresponding *Y*_H_2__ were calculated using a permeate pressure of 0.2 bar with real separation energy and *η*_s→e_ of 15% for a typical sunny day in Beijing [50] during each of the four seasons (Figure 11). The change in *Y*_H_2__ is similar to that in *DNI* because the obtained energy amount decides the available heat for ESR reaction. As discussed above, reacting at higher temperatures (e.g., 300 °C) results in high ESR conversions but does not coincide with the highest *η*_s→f,real_ due to the radiation loss. The variation in *Y*_H_2__ is similar to that in *η*_s→f,real_, and the highest *Y*_H_2__ occurs at 200 °C. The highest *Y*_H_2__ of 22.4 mol·m^−2^·h^−1^ at noon occurs in June with the strongest radiation levels, whereas the highest *Y*_H_2__ during December is 16.6 mol·m^−2^·h^−1^ at noon. The total generation is also affected by the comparatively longer working hours in June versus the reduced working hours in December. Assuming that the annual amount of solar radiation is 2000 h [51,52], the annual *Y*_H_2__, SCSR, and CDRR are estimated to be 72.6 kmol·m^−2^·y^−1^, 201.38 kg·m^−2^·y^−1^, and 493.40 kg·m^−2^·y^−1^, respectively. Notably, however, the temperature does not have a significant effect on the overall performance above 150 °C.

**Figure 12:**
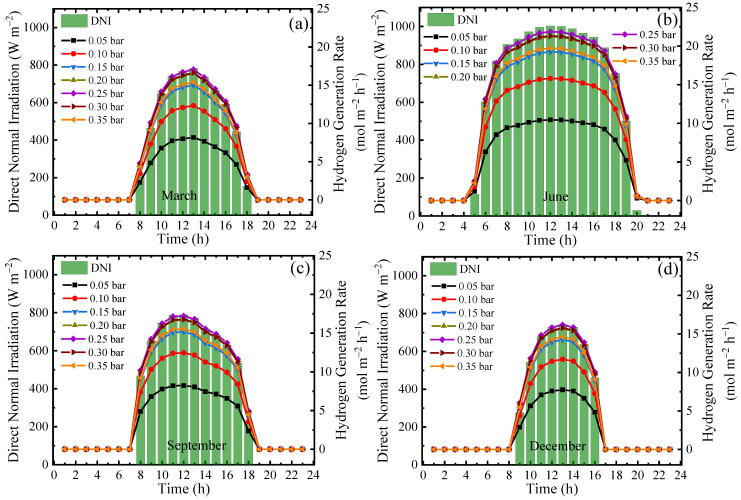
Variation in *DNI* and *Y*_H_2__ on a typical sunny day of different months for different permeate pressures at a reaction temperature of 150 °C with real separation energy (*η*_s→e_ = 15%): (**a**) March; (**b**) June; (**c**) September; and (**d**) December.

Compared with temperature, the permeate pressure has a more significant effect on the generated *Y*_H_2__ for each of the seasons. The results for generated *Y*_H_2__ are shown in Figure 12 for a reaction temperature of 150 °C with real separation energy and *η*_s→e_ of 15%. The optimum permeate pressure for *Y*_H_2__ is 0.25 bar, which produces a *Y*_H_2__ of 21.9 mol·m^−2^·h^−1^ at noon in June. The results shown in Figure 10 and Figure 11 exhibit the promising application of the solar-driven ESR system under realistic operating conditions.

## 5. Conclusions

A novel solar-driven ESR system integrated with a membrane was proposed for solar energy storage and hydrogen production. This concept presents a promising approach for increasing conversion efficiency and has great potential for being utilized in distributed energy systems. The thermodynamic and environmental performances were analyzed, with the following highlighted conclusions:

(1) An ESR system can improve the conversion rate at relatively low temperatures due to simultaneous separation of hydrogen. The theoretical conversion rate of the membrane reactor at 100 °C with permeate pressure of 0.01 bar is 98.3%, compared with only 14.8% for a traditional reactor configuration.
(2) Solar energy can be utilized efficiently in this system. The first-law thermodynamic efficiency, solar-to-fuel efficiency, and exergy efficiency with separation exergy and *η*_s→e_ of 40% are 82.3%, 45.3%, and 70.4%, respectively, at 215 °C and 0.20 bar. Compared with a traditional reactor, the *η*_HHV,real_ achieves the largest increment and can reach 12.0% at 130 °C with *η*_s→e_ of 40%.
(3) In terms of the environmental performance of this solar-driven ESR system, higher efficiency leads to less fuel consumption and lower CO_2_ emissions. The SCSR and CDRR can achieve maximums of 101 g·m^−2^·h^−1^ and 247 g·m^−2^·h^−1^ at 200 °C and 0.20 bar with a corresponding hydrogen generation rate of 22.4 mol·m^−2^·h^−1^. The annual SCSR and CDRR are expected to be 201 kg·m^−2^·y^−1^ and 493 kg·m^−2^·y^−1^.

The proposed ESR membrane reactor provides insight into a means of efficient storage and conversion of solar energy, with potential for utilization in small energy systems, such as refueling stations for hydrogen fuel cell vehicles.

## Data Availability

The data presented in this study are available on request from the corresponding author.
